# Radiomics As Biomarkers for the Treatment of Non-small Cell Lung Cancer With Stereotactic Body Radiation Therapy: A Review of Concepts

**DOI:** 10.7759/cureus.73082

**Published:** 2024-11-05

**Authors:** Gemini Ramasamy, Thierry Muanza

**Affiliations:** 1 Experimental Medicine, McGill University, Montreal, CAN; 2 Radiation Oncology, Sir Mortimer B. Davis Jewish General Hospital, Montreal, CAN

**Keywords:** biomarkers, management, non-small cell lung cancer, radiomics, stereotactic body radiation therapy

## Abstract

Stereotactic body radiation therapy (SBRT) is currently the alternative for inoperable early-stage and oligometastatic non-small cell lung cancer (NSCLC) patients. While most patients are good responders among this specific group, some patients do not experience the benefits of this treatment. Even though physicians use clinical variables and semantic radiological features to make treatment decisions, medical images contain a wealth of personalized pathophysiological information that can be extracted and used for clinical decision support systems. In the form of radiomics features, details unique to each patient's medical scans can be utilized to create predictive models and to identify biomarking signatures. Then, these tools and indices can predict treatment outcomes and categorize patients to the most optimal treatment regimen.

A conceptual review of relevant topics centered around the identification and development of radiomic-based biomarkers for SBRT-treated NSCLC was conducted. To begin with, an overview of the nature and management of non-small cell lung cancer was provided. To continue, biomarkers were defined in the context of cancer care. Then, the uses of stereotactic body radiation therapy in the treatment of NSCLC were further explained. Finally, the study of radiomics was discussed, and the uses and limitations of radiomic features and ML for SBRT-treated NSCLC were expanded upon.

Radiomics-based biomarkers and predictive algorithmic models can potentially improve the SBRT treatment of early-stage and oligometastatic NSCLC by providing personalized support systems to healthcare professionals. While many institutions are attempting to optimize their biomarkers and AI-based tools for clinical use, additional prospective studies are needed to properly ensure their efficacy. As such, the improvements made in the field of personalized medicine are promising.

## Introduction and background

Brief introduction

In recent years, artificial intelligence (AI) has been changing the way we live; it is increasing speed and efficiency when performing common tasks. AI has also allowed the creation of various useful support systems. One area that has benefited from AI support is radiation oncology. Indeed, the field of oncology has achieved new heights; great strides have been made in fundamental, translational, and clinical research. This is especially true when we consider our handling of medical information and our approach when learning from data. As radiation oncology requires multiple scans and clinical information for disease assessments and treatment decisions, the amount of potential data collected is massive. It is only natural to study this data to better understand diseases. Using artificial intelligence, such as machine learning (ML), researchers have been working on different ways of harnessing the power of high-dimensional computing techniques to gain the most from data available on cancer patients.

Lung cancer is the most significant source of cancer-related deaths worldwide [1,2]. Specifically, non-small cell lung cancer (NSCLC) is the main type of lung tumor [1]. This lung cancer subtype has amassed a large amount of data; this is mostly due to the visibility of solid NSCLC tumors with medical scans, especially computed tomography (CT) scans, the high volume of lung cancer patients, and the widespread reach of the disease, due to the smoking of tobacco. Since the research and development of AI requires an immense quantity of varied data, artificial intelligence can truly showcase its full potential in NSCLC research.

Depending on the cancer progression stage, NSCLC patients can receive different treatments. In the case of early-stage NSCLC patients, surgical resection is the standard treatment [3]. However, if the patient has medical complications morbidities, or refuses to receive surgery, stereotactic body radiation therapy (SBRT) is the standard alternative treatment [3-5]. SBRT is a type of external beam radiotherapy treatment; it uses advanced treatment planning techniques to accurately, precisely, and stereotactically target parenchymal lung tumors, all the while minimizing any radiation exposure to surrounding, healthy tissues or any critical, thoracic structures [6].

In addition, over the years, tomographic images, such as CT, MRI, and PET, have been increasingly showcasing their full potential. With the help of various machine learning processes for quantitative feature extraction and pattern recognition models, medical images have been transitioning to become mineable data. Indeed, beyond human-made observations, radiomics has emerged as the practice of extracting objective features from medical imaging sets. Similarly to its other "omics" counterparts, radiomics accumulates data to provide specific, unique, and almost imperceptible information about individual patients. Actually, the field of radiomics attempts to reduce the subjectivity of visual interpretation by providing quantifiable features and by associating these features with pathophysiology. Hence, radiomic models, in conjunction with patients' data and multi-omics, could be used as highly accurate, clinically relevant diagnostic/prognostic/predictive tools. Such biomarkers and models could bring healthcare closer to precision medicine [7,8].

In this conceptual review, the topics of biomarkers and radiomics in the context of SBRT-treated NSCLC will be discussed. First, we will describe lung cancer as a disease and how it is treated and managed. Second, we will define the nature of biomarkers. Also, we will describe biomarkers' uses in the context of cancer. Third, SBRT and its' role in NSCLC treatment will be described. Fourth, we will delve deeper into the topics of radiomics and ML, and we will discuss how radiomics and ML contribute to the treatment of NSCLC patients with SBRT. Furthermore, we will acknowledge the achievements made in the field up until now. Finally, the limitations of machine learning and radiomics will be discussed. This review will cover the topics required to conduct research on radiomics-based biomarkers for SBRT-treated NSCLC patients.

## Review

Lung cancer

Introduction to Lung Cancer

Lung cancer has been among the top-ranking causes of cancer deaths in men and women for years worldwide [9]. Indeed, this malignant tumor formed from the epithelial cells of the lung parenchyma or within the bronchi has caused a high death toll on the human population [10]. While general cancer incidence depends on age most of the time, external factors constitute a risk of specifically developing lung cancer [11]. Many of these risk factors have embedded themselves in the culture and habits of people, such as smoking tobacco [11].

Lung cancer develops through a multistep process in which mutations and epigenetic changes create defects in the regulation of proliferation and homeostasis [12]. Eventually, the accumulation of such defects transforms normal cells into malignant cells. As the cells proliferate uncontrollably through clonal expansion, the cancer becomes more invasive, and it spreads to the lymph nodes and distant organs [12]. There are two main histological types of primary lung cancers: non-small cell lung cancer (NSCLC) and small cell lung cancer (SCLC) [13]. While both types are characterized by the uncontrolled cell growth of malignant tumors in the lung's epithelium, NSCLC has larger and slower proliferating cells than SCLC, which displays much smaller cell sizes [12]. For this conceptual review, there will be a focus on NSCLC, as it constitutes 85% of lung cancers [14].

General Overview of Management of NSCLC Patients

NSCLC management differs based on the cancer staging, among other variables [15,16]. When dealing with stage I patients, surgery is often the preferred option. If the patient cannot receive surgery - due to comorbidities or poor lung function - or refuses the intervention, stereotactic body radiation therapy (SBRT) can be used instead. Without lymph node involvement, stage II patients receive the same types of treatments as stage I. Although, with node involvement, SBRT is not an option, and full-dose conventional fractionation radiation therapy (RT) needs to be given instead [13]. In other words, stage I and stage II NSCLC, without node involvement and without mediastinum invasion, are considered early-stage NSCLC [13]. This classification is important for our review because early-stage NSCLC patients can receive SBRT as an alternative, definitive treatment [13]. Additional chemotherapy and immunotherapy are also considered, depending on the situation [13].

As the staging increases to III, combined therapy is used; either a combination of surgery and chemotherapy, or a combination of radiation and chemotherapy, along with targeted therapy or immunotherapy, are possibilities [17]. Additionally, adjuvant chemotherapy can be given after the main treatment regimen [13,17]. Finally, with palliative intent, stage IV patients can receive most of the procedures previously mentioned, except for surgery [15,16,18]. In contrast with early-stage NSCLC, advanced NSCLC includes stage III and stage IV. Specifically, stage III NSCLC is considered a locally advanced disease, and stage IV NSCLC corresponds to a disseminated, advanced disease [13]. Advanced stage IV NSCLC specifically includes stage IV patients and patients with disease recurrence after an initial, definitive treatment has been given [19]. Generally, for stage I, II, and III NSCLC, the treatments given are meant to be curative [19]. Meanwhile, stage IV NSCLC receives palliative treatments, except for oligometastatic NSCLC [19].

Managing Stage I NSCLC

When treating stage I patients, surgery is often the preferred option. Specifically, to treat a stage IA or IB peripheral tumor, a lobectomy can be performed. In contrast, a central tumor requires a multidisciplinary approach; the addition of chemotherapy to the treatment regimen can be beneficial for the patient. Neoadjuvant or adjuvant chemotherapy can be given alongside the surgery [13]. For patients who receive surgery first, pathologic staging can be assessed using the tumor tissue resected. As stated in Valliere et al.'s review, while adjuvant chemotherapy is not recommended for pathologic stage IA patients, pathologic stage IB patients with high-risk features can receive adjuvant chemotherapy [13]. Additionally, surgical stage I patients with positive margins can receive postoperative radiation therapy. Positive margins indicate that cancer was found in the surgical margins of the resected tissue; this finding suggests that cancer was still present at the resection site [13].

If a patient cannot receive surgery - due to comorbidities or poor lung function - or refuses the intervention, definitive radiation therapy, such as SBRT and full-dose conventional fractionation RT, can be given as an alternative [13]. As mentioned in Valliere et al.'s review, SBRT is recommended for tumors smaller than 5 cm [13]. As for tumors larger than 5 cm, curative intent intensity-modulated radiotherapy (IMRT) with or without gating is the recommended treatment option [13].

Managing Stage II NSCLC

Similarly to the management of stage I NSCLC, surgical resection is the main treatment recommended for stage II NSCLC. Additionally, neoadjuvant induction chemoimmunotherapy is suggested for clinical stage II NSCLC. In general, for stage II patients, the combination of nivolumab and neoadjuvant, platinum-based chemotherapy has shown benefits [13]. On the other hand, if surgery is given first, pathology can be assessed; if a patient has pathologic stage II NSCLC, adjuvant chemotherapy can be recommended [13]. Also, postoperative radiation therapy can be given to surgically treated stage II patients with positive margins [13].

Furthermore, if a patient cannot receive surgery - due to comorbidities or poor lung function - or refuses the intervention, definitive radiation therapy can be used as an alternative [13]. When the stage II NSCLC has not spread to lymph nodes, the choice of radiation therapy technique follows the same guidelines as for stage I patients [13]. However, with node involvement, SBRT is not an option for stage IIB NSCLC, and definitive, standard-fractionation radiation therapy is given instead of SBRT [13]. Moreover, chemotherapy and immunotherapy may be added to the treatment regimen to provide support, depending on the situation [13].

Lastly, it is important to note that stage I and stage II NSCLC, without node involvement, are interpreted as early-stage NSCLC [13].

Managing Stage III NSCLC

As the extent of NSCLC increases to stage III, the affected population becomes very heterogeneous, specifically pertaining to tumor sizes, invasion of lymph nodes and surrounding structures, and localization of disease [17]. Consequently, combined therapy is used to manage many different disease configurations. Possibilities include a combination of surgery and chemotherapy or a combination of radiation and chemotherapy, along with targeted therapy or immunotherapy. For instance, the phase III PACIFIC trial showed that giving immunotherapy after concurrent chemotherapy in locally advanced, stage III NSCLC increased the patient's five-year probability of overall survival from 33.4% to 42.9% [20]. Additionally, during stage III NSCLC, adjuvant chemotherapy can be given after the main treatment regimen [17].

Generally, to determine the proper treatment regimen for stage III NSCLC, the mediastinal lymph nodes need to be assessed pathologically for tumor involvement [17]. If the mediastinal lymph nodes are not involved, either induction chemotherapy followed by surgery or perioperative chemoimmunotherapy and surgery can be given to EGFR/ALK-negative patients. For EGFR/ALK-positive patients, a surgical resection followed by adjuvant systemic therapy can be given [17]. On the other hand, if the cancer reaches the mediastinal lymph nodes, concurrent chemoradiation - usually platinum-based chemotherapy with full-dose radiation therapy - is suggested, with the possibility of adding another treatment modality to the regimen [17].

Managing Stage IV NSCLC - Advanced Disease

Advanced NSCLC management focuses on a palliative approach; the objectives are to improve the patient's progression-free survival, maintain a good quality of life, and reduce the side effects of treatments [19]. Hence, the treatments given for advanced NSCLC are focused on palliation, not curation. This approach does not use definitive treatment modalities. This treatment intent affects the aggressivity of treatments and the reasoning for using specific therapies [19].

Usually, systemic treatment is given to patients with advanced disease NSCLC [17]. Systemic therapies are given concurrently or sequentially; therapies include immunotherapy, cytotoxic chemotherapy, biologics, and targeted agents [19]. The initial therapy is determined by variables such as histology, driver mutations, PD-L1 expression, and patient-specific factors [19].

Non-squamous, advanced NSCLC should be tested for driver mutations such as epidermal growth factor receptor (EGFR), anaplastic lymphoma kinase fusion oncogene (ALK), and more [19]. Indeed, chemotherapy is not the first-line therapy for stage IV patients with targetable mutations. When the tumor has a driver mutation, the suggested therapy is to use a specific inhibitor for that driver mutation until progressive disease is observed [19]. Conversely, when the tumor does not have driver mutations, the expression level of PD-L1 is used to determine the proper course of treatment. The expression of PD-L1 on the cell surface is a marker that predicts the response of immune checkpoint inhibitors [21]. For high PD-L1 expression tumors (tumors with at least 50% of cancer cells expressing PD-L1), pembrolizumab or atezolizumab monotherapy is given [19,22]. That said, for patients with rapidly progressive disease and high tumor burden, concurrent chemotherapy and immunotherapy are given [19]. For lower PD-L1 expression levels, a combined approach using chemotherapy and immunotherapy has shown to improve survival [21]. Lastly, to manage specific metastatic sites, additional treatments can be used to control symptoms caused by the metastases [19].

Managing Stage IV NSCLC - Oligometastatic Disease

There are some stage IV NSCLC cases in which the number of metastases is limited to one or only a few. This oligometastatic state describes patients with metastases limited in number and organ sites [23]. Specifically, the European Consensus definition states that oligometastatic disease has up to five metastases from three sites [24]. Systemic therapy can potentially eliminate these limited, small oligometastases [23]. Indeed, as treatments are becoming more refined, it may be possible to achieve substantially longer overall survival and to cure patients of cancer. For oligometastatic NSCLC patients, there are potential benefits in using more aggressive and definitive treatment modalities to target oligometastases [23].

When patients with one to three oligometastases have a good performance status, local therapy, such as surgical or ablative treatments, can be given to all oligometastatic sites alongside systemic therapy. That said, the decision to pair definitive treatments with systemic therapy must be done in a multidisciplinary setting [23]. Furthermore, the choice of systemic therapy follows the same management guidelines as for advanced NSCLC treatment [23]. As for local therapy, surgery, and radiotherapy, such as SBRT, can be given, but nonoperative techniques are preferred for patients with debilitating comorbidities [23].

Currently, the optimal order of definitive and systemic treatments is not known, and it is mainly theorized [23]. On the one hand, giving systemic therapy first may allow the confirmation of the patient's stage IV oligometastatic disease, thus indicating that local ablative therapies may be beneficial. On the other hand, giving local ablative therapies followed by systemic therapies may be considered for cases of oligorecurrence/oligoprogression [23]. In this specific case, systemic therapies would only be given when there is radiographic evidence of widespread metastatic disease after the local therapy [23].

Lung Cancer Conclusion

The treatment of lung cancer is a multifaceted process that can proceed in various ways. Indeed, depending on the symptoms, the staging, the imaging, the tumor histology, the molecular profiling, the genetic alterations, and the patient's wishes, a combination of different therapies can be recommended. The recommended treatments include surgery, radiotherapy, chemotherapy, and various targeted therapies. By using these different approaches, clinicians attempt to stop cancer progression, reduce tumor volumes, improve overall survival, minimize the risk of recurrence, palliate the toxicity/side effects experienced, and offer a good quality of life to patients.

Cancer biomarkers

Introduction to Cancer Biomarkers

With the shift from empirical to personalized medicine in recent years, there has been a need for specific characterization tools. Indeed, it is no longer enough to base clinical decisions on symptoms or to follow generic, systemic decision processes when selecting treatment options. To create personalized treatment regimens, there is a need for variables capable of providing an in-depth understanding of patients' conditions.

Biomarkers are quantifiable and objective characteristics that can be measured to assess the state of a patient and to formulate predictions or prognoses [25]. The nature of biomarkers can vary from DNA, RNA, protein, and peptides to post-translational modifications, metabolomics, radiomics, etc. Actually, there has been an increasing interest in quantifying and targeting pathways at the molecular level due to advances in molecular biology, the rise of high-throughput screening, and molecular characterization of tumor tissue [25]. As advances in multi-omics are progressing and our knowledge of cancer biology is increasing, a new generation of clinically relevant biomarkers is needed to support decision-making in therapeutics and prognostics [25].

More specifically, biomarkers can help to identify populations for which a drug/treatment is very effective. The use of such biomarkers can result in more successful clinical trials. Indeed, in regular trials, more than 90% of oncological drugs that enter clinical development do not reach approval for phase IV trials, which is an observational phase conducted post-approval to identify less common adverse reactions and to evaluate cost and drug effectiveness in larger populations. [26] because earlier trial phases fail to show a clear therapeutic benefit [27]. To decrease the risk of trials and the number of clinical trial failures, we can use biomarkers to identify the molecular subtypes that would benefit the most from potential drugs/treatments. Furthermore, biomarkers have the potential to improve the healthcare system in many ways. Currently, many patients are taking drugs without benefits [25]. Biomarkers can help to define the heterogeneity in drug response between patients. Lastly, anti-cancer drugs are expensive and toxic; biomarkers can prevent their unnecessary use [25].

Cancer Biomarkers in Clinical Settings

In terms of clinical use, cancer biomarkers can be used to evaluate the risk of cancer development, the cancer progression, and the response to therapy and procedures [25]. Specifically, these applications are useful when cancer biomarkers are linked to specific molecular pathways in cancer pathogenesis. Indeed, by linking the fluctuations in biomarker presence and/or levels to molecular pathways, the use of specific interventional strategies can be justified [25].

In clinical settings, different types of cancer biomarkers have their practical uses. Here are a few types described by Goossens et al. [25]. Predictive biomarkers predict the response/outcome to specific treatments/interventions. For example, HER2 positive can predict the response to trastuzumab treatment in breast cancer [28]. Also, in colorectal cancer, KRAS-activating mutations can predict EGFR inhibitor resistance [29]. While prognostic biomarkers are not useful for making therapeutic decisions, they can predict key clinical outcomes, such as cancer recurrence, disease progression, and survival. Lastly, diagnostic biomarkers are used to diagnose patients and to determine if patients have specific conditions. For example, the presence of cancer DNA in stool would indicate the presence of colorectal cancer [25,30].

Cancer Biomarkers Development

To develop a cancer biomarker, a relationship must be established between an observable characteristic of the tumor, the patient or treatment mechanism, and the tumor biology. Indeed, cancer biomarkers are developed by identifying and quantifying key features directly linked to a tumor's biological processes.

There are various types of cellular biomarkers, including DNA, RNA, and proteins. In the case of DNA biomarkers, DNA alterations, such as amplifications, deletions, point mutations, and gene fusions, can be used as biomarkers [25, 31]. To illustrate, if a drug targets a specific disease-activating, mutated gene, the targeted gene would be an ideal biomarker. The presence of the targeted gene might indicate the potential effectiveness of the drug [25, 27]. While using DNA as a basis for biomarkers is useful due to its consistency, screening DNA for biomarkers is time-consuming [25].

Regarding RNA biomarkers, there are advantages and disadvantages of measuring specific RNA levels. While it is a chemically unstable molecule that fluctuates in concentration, RNA is more accessible to measure. Compared to DNA, which is mainly located in the nucleus, RNA carrying precise genes can be measured in the cytoplasm [25]. Although one single RNA molecule does not give interpretable information, the concentrations of many different RNA strands can easily be measured at the same time [25].

Furthermore, the proteome offers the maximum level of complexity because they are the effectors of cellular systems. Despite the fact that proteins are very unstable and difficult to measure reliably, they display post-translational modifications, such as glycosylation, oxidation, and phosphorylation [31]. Protein-based biomarkers can take advantage of this level of cellular complexity to characterize the tumor and its environment [25, 31].

Moreover, even though the previously mentioned biomarkers are profiled with the intention to understand the primary tumor, there has been some evidence showing that metastatic tumors are different compared to primary tumors [12, 13, 19]. Inter-tumor and intra-tumor heterogeneity are complexities of cancer treatments [31]. The clinical decisions based on site-specific biomarkers cannot be generalized to the entire system due to potential variability [31].

With the advent of high-throughput methods, it is possible to combine different research fields, such as genomics, transcriptomics and proteomics, and create biomarkers based on multi-omics. This multidisciplinary approach to finding cancer biomarkers enables a multifaceted characterization of patients, which may bring healthcare closer to precision medicine. Also, different omics fields, such as radiomics, can provide a completely different set of information. As radiomics is image-based, using this field in biomarker discovery can give an anatomical and histopathological dimension to biomarkers [7].

While there are many obstacles preventing the comprehensive study of patients, including the cost, the feasibility of a clinical setting, and the involvement needed from patients, the increasingly multidisciplinary nature of cancer care is advantageous for the development of biomarkers.

Cancer Biomarkers Limitations

While biomarkers are very useful, there are limitations to their development and implementation. Indeed, when developing and implementing biomarkers, there are issues concerning invasiveness. Most of the time, to measure DNA, RNA, and protein-based biomarkers, the tumor needs to be biopsied, which can be too invasive for some patients. On the other hand, radiomics-based biomarkers are less invasive, as they only require medical images. Additionally, the quality of the tumor tissue, the timing of tissue collection, and the tumor heterogeneity introduce intra-patient and inter-patient variability.

Furthermore, while multiple reports on highly performing biomarkers are being published, only a few biomarkers are clinically implemented [32, 33]. Implementing biomarkers in a clinical setting is challenging due to three main reasons. Firstly, there is a difference between analytical validity and clinical validity. Initially, many discovered biomarkers have high sensitivity, specificity, and statistical significance when tested in vitro; then, the biomarkers are qualified as analytically valid. That said, when these biomarkers are clinically implemented, they are found to be clinically unhelpful. Consequently, these analytically valid biomarkers have no clinical validity. The lack of clinical validity may be caused by the weakness of the information provided by the biomarker, the biomarker's lack of relevance for decision-making, or the biomarker's low efficiency in a clinical setting [32, 33].

Secondly, the positive predictive value (PPV) and the negative predictive value (NPV) may not be above the defined clinical threshold. The PPV is the probability that a patient has a disease/condition when the test is positive [34]. The NPV is the probability that a patient does not have a disease/condition when the test is negative [34]. Even though some studies address unmet clinical needs, some biomarkers are not implemented due to the risk involved in using them. For example, using a biomarker with a low PPV could potentially classify a patient as positive, even though the patient might not actually have the condition/disease. Such a mistake could cause a patient to receive unnecessary, and perhaps harmful, treatments [34]. Also, during development, obtaining a high PPV and NPV can be challenging due to the expensive, time-consuming, and large studies required to establish clinical value [25, 27, 34]. Additionally, when studying biomarkers for rare cancers, the potential for positive results is low; in this case, the PPV and NPV can become distorted [34].

Lastly, biomarkers must demonstrate clinical utility; in other words, biomarkers must have the potential to change the current clinical practice. If a biomarker does not impact clinical outcomes or decision-making, the biomarker's purpose is unclear [25, 27]. For instance, biomarkers should allow a clinician to help a patient in the following ways: decreasing toxicity, increasing overall survival (OS), decreasing time to progression (TTP), increasing progression-free survival (PFS) or increasing quality of life (QOL) [25, 27].

Other limitations in cancer biomarker development and implementation include issues in study design, issues in assay platforms, the lack of inter-lab reproducibility, technical issues, the lack of relevance of pre-clinical models, the difficulty in obtaining tissue samples in sufficient quantities, and the need to conduct expensive clinical trials for prospective validation [32,33].

Cancer Biomarker Conclusion

While it is challenging to bring new biomarkers to clinical use, there are promising biomarkers for lung cancer. In terms of NSCLC biomarkers, specific radiomic features (objective data extracted from medical images) were shown as prognostic for distant metastases in lung adenocarcinoma [35], disease recurrence [36], and overall survival [37]. Also, some radiomic features were determined to be predictive of treatment response. For example, first-order skewness and root-mean-squared-intensity were shown to be predictive of local response for SBRT-treated NSCLC tumors [38].

Cancer biomarkers have the potential to improve cancer treatments by predicting treatment outcomes, formulating more accurate prognoses, and supporting clinical decisions. Essentially, biomarkers are employed to decrease the morbidity of treatments and to improve treatment outcomes, such as overall and progression-free survival. Ultimately, biomarkers could prevent unnecessary suffering, thus putting the overall well-being of patients on the forefront.

Stereotactic body radiation therapy

Stereotactic Body Radiation Therapy Definition

Stereotactic body radiation therapy (SBRT) is a type of external beam radiotherapy. SBRT uses advanced treatment planning techniques to accurately and precisely target extracranial tumors while minimizing any radiation exposure to surrounding healthy tissues or critical thoracic structures [6,39]. Using a linear accelerator paired with the appropriate image-guidance technology, SBRT delivers a high dose of radiation (larger than 6 Gy per fraction) [6] in a single or a few fractions to a small, well-defined lesion at a high degree of accuracy and precision [40].

To achieve this level of targeted delivery, stereotactic localization of the tumor and tumor-site-specific image guidance are required to locate and delineate the target volume and to minimize the margins of the dose delivery. Indeed, the target is clearly separated from the nearby organs at risk; only the target tumor and its adjacent, microscopic tumor spread receive radiation [39]. For stereotactic localization of tumors, stabilizing apparatuses, such as body frames, vacuum cushions, thermal plastic restraints or featured stereotactic fiducial systems [6,41], are used to ensure a stable target tumor by positioning and immobilizing the patient. Once the patient's body is correctly placed, the tumor motion is reduced with controlled abdominal compression, deep inspiration, respiratory gating or tumor tracking systems [6,41]. All in all, SBRT is conducted by following a highly optimized workflow, requiring multiple sophisticated techniques and quality assurance measures [39].

SBRT Treatment for Lung Cancer

As for SBRT's clinical applications, it is currently established as the standard treatment alternative for early-stage NSCLC patients who are not appropriate candidates for surgery or refuse surgery altogether [39,42-44]. That said, some aspects of SBRT are still being improved upon, and new research directions are being explored. With many trials and retrospective studies about SBRT, researchers are trying to examine the nuances of this treatment. For instance, the multicentric, phase III, randomized CHISEL trial (NCT01014130) demonstrated that using SBRT on medically inoperable stage I patients was associated with better local control [45]. Indeed, local failure rates after two to three years were reduced when patients with peripheral tumors received SBRT (14%) instead of conventional, fractionated radiotherapy (31%). Also, an improvement in overall survival was shown. After two years, the overall survival for SBRT patients was 77%, while conventional fractionated therapy's rate was 59% [45]. While SBRT has gained in popularity because of high local control rates, there is still a tendency of recurrence as developing distant metastases. For example, in the Radiation Therapy Oncology Group, phase II, non-randomized, multicentric study (RTOG 0236), 55 medically inoperable, early-stage patients were treated with SBRT [46,47]. While the three-year local control (focused on the tumor and the involved pulmonary lobe) rate was at 90.6% and the local-regional control rate was at 87.2%, the three-year rate of disseminated failure (failure beyond local and regional sites) was at 22.1% [46,47].

Furthermore, SBRT has been shown to be effective against oligometastases within the lung. The oligometastatic state designates stage IV NSCLC patients with a limited spread of microscopic metastases constrained to a small number of sites, ranging from one to five oligometastatic lesions. These lesions follow this specific development pattern because of anatomical and physiological factors [48]. Some specific patients can benefit from a curative treatment approach to a single or a few lung oligometastases. For example, in the randomized, multicentric, phase II SABR-COMET trial, 99 stage IV patients were enrolled to receive either palliative, standard-of-care treatments or standard-of-care treatments and SBRT for all the metastatic lesions [49,50]. The patients had one controlled primary tumor (not specifically NSCLC) and one to five oligometastatic lesions. The overall survival rate after five years for the SBRT-treated group was 42.3%, while the group receiving only palliative treatments had a rate of 17.7%. As for the overall, long-term (five years) local control, the rates were 63% in the SBRT-treated group and 46% in the palliative treatments group [49,50]. Traditionally, the treatment of choice for a single and small oligometastasis is surgical resection, but SBRT appeared to be an effective and well-tolerated, non-invasive local therapy for patients with limited, metastatic disease within the lung. Thus, SBRT is an alternative to surgery for oligometastatic patients [49,50].

In the case of medically operable NSCLC patients, using SBRT for treatment instead of surgery is not as straightforward. Many randomized trials have attempted to compare the benefits of SBRT versus surgery, but the trials could not reach full completion. Indeed, the phase III randomized STARS (NCT00840749) and ROSEL (NCT00687986) trials were both closed early due to slow accruals, thus preventing the determination of a conclusive advantage for one treatment [51]. Later, a pooled analysis of the trials was conducted to combine and interpret the results. Together, the pooled trials assigned 58 operable, stage I NSCLC patients to receive either SBRT or surgery. While the three-years overall survival rate was 95% for the SBRT-treated group, the surgery-treated group had a rate of 79%. In comparison, for the three-years recurrence-free survival, the difference between the rates of the SBRT-treated group (86%) and the surgery-treated group (80%) was not as large. Even if SBRT seemed to have potential, this pooled analysis had a low sample size and a short follow-up [51]. To find a conclusive answer, research groups are conducting further studies. For example, STABLE-MATES (NCT02468024) and VALOR (NCT02984761) are current trials comparing stage I patients treated with SBRT or surgery [52].

Radiomics

Artificial Intelligence and Machine Learning

Artificial intelligence (AI) is a broad term that describes computational algorithms and frameworks capable of analyzing and learning from large datasets. Generally, AI can learn how to classify data, how to predict the rest of a dataset, how to create new data and how to make decisions. AI learns from datasets, which means that the larger and the more diverse these datasets are, the better an AI will be at accomplishing tasks [7,8]. Additionally, the quality of the datasets is crucial; if datasets have too many errors or biases, AI will learn from mistakes, and will perform tasks poorly. As for machine learning (ML), it is encompassed in the definition of AI; ML designates the creation and the adjustment of statistical models based on datasets. ML learns from datasets by calibrating a selected statistical model through training and testing. Also, ML can apply statistical processes on data to extract useful features [7,8].

Radiomic Features

In the field of radiomics, there are two general types of features: semantic features and agnostic features. While semantic features, such as diameters, shape, volumes, and vascularity, are frequently used by radiologists, agnostic features, such as gray-level histograms and textures, are more subtle, and they require computational assistance to be fully appreciated [7]. There are various technical classes of radiomic features: histogram features, texture features, model-based features, transform-based features, and shape-based features [7,8].

Histogram features are statistical descriptors that examine images, pixel by pixel, or voxels by voxels [8]. The global gray-level histogram is commonly used in medical imaging; this histogram shows the amount of pixels with specific gray-levels. While the x-axis displays a gradation of gray-levels, the y-axis shows the relative amounts of pixels with distinct gray-levels. Based on this histogram, values, such as the gray-level's mean, maximum, minimum, variance, and percentiles, are observed, and they are used for analyses. Data distribution features are also included. Indeed, while skewness describes the left/right asymmetry of a data distribution, kurtosis describes any outlier-caused tailedness in a data distribution when compared to a Gaussian distribution [8].

Textural features describe the relationships between pixels based on the gray-levels [8]. Using an absolute gradient is a simple approach to describe texture; the gradient describes the extent of gray-level fluctuation between two adjacent pixels/voxels. Either a positive gradient (from darker to lighter levels) or a negative gradient (from lighter to darker levels) is assigned to every adjacent pixel/voxel [8].

Model-based features characterize the spatial gray-level to distinguish the borders of an object or shape [8]. Essentially, a parameterized model is generated and fitted to the region of interest (ROI) of an image based on spatial gray-level textures. Then, the optimal parameters found for the ROI of an image are reported as features [8].

Transform-based features use wavelet transform methods to observe gray-level patterns at different scales and directions [8].

Shape-based features characterize geometric properties within an ROI; observed features include diameters, axes, dimensional ratios, compactness, and sphericity [7,8].

Applications of Radiomics and ML for SBRT-Treated NSCLC Patients

There have been many potential applications of AI for the treatment of lung cancer with SBRT. For example, there are machine learning algorithms trained to delineate and contour solid tumors from CT, PET, and MRI scans. As the tumor delineation varies with the radio-oncologist responsible for a patient, an AI capable of accurately contouring a tumor would be very useful in a clinical setting. Such an algorithm could potentially reduce inter-observer discrepancies, and it could contour tumors more consistently. Furthermore, radiomics and deep learning have been used to discover prognostic and predictive biomarkers. These biomarkers could have multiple uses in the case of cancer prediction. For instance, the biomarkers could be used to generate a prognosis of the tumor and predict the overall progression of the disease based on scans, clinical features, and patient characteristics. Specifically in the case of SBRT-treated, NSCLC patients, machine learning models trained with radiomic features from pre-treatment planning CT scans were able to predict local control [53], recurrence [54], the development of distant metastases [55], survival [56] and radiation-induced side effects to the healthy lung tissue [57-59].

While there are tools for tumor control probability (TCP) prediction based on the prescribed SBRT radiation dose, which is then corrected into the biologically effective dose (BED) to account for different tumor histology and fractionation schemes [60,61], only a few tools take advantage of the data available in medical imagery, using radiomics or deep learning [60,61].

Predictive models can also contain various modalities of prediction. Indeed, the addition of other variables to predictive models, such as clinical variables, has been a research direction recently explored in the field of predictive, algorithmic models. For example, in Avanzo et al.'s study, they combined radiomic and deep learning features with the BED of the SBRT to create models that predicted the response of patients' lung cancer to their SBRT treatment [59]. They found that both deep-learning-based models and radiomics-based models have better performances when combined with the BED compared to models without the BED [59].

Limitations

While the field of radiomics is expanding, there are limitations that prevent the use of radiomic features in clinical settings. To begin with, when developing a radiomics-based model, the images used must be of high quality and artifact-free; the model must be trained with obstruction-free, clear images. Furthermore, there is a lack of standardization when acquiring medical images. Indeed, the acquisition and reconstruction methods are not homogenous from one healthcare site to the next [46,47]. These oversights cause a lack of reproducibility for the models' performance. For instance, a model might perform well at the training site, but when it is deployed to another site, the model's performance might suffer due to different image resolutions. Then, model and biomarker validation is needed to ensure stability across institutions [7]. Additionally, this example emphasizes the need for a crucial balance in fitness in radiomics-based models. Fitness occurs when a model has been trained so closely to its dataset that the model considers very specific details when making decisions [8]. An ideal model should not be overfitted nor be underfitted; a model should be generalizable enough to accommodate systemic differences in images and various ROIs. That said, generating an ideal model is a challenge.

Moreover, class imbalance is another example of a problem encountered when training a model, especially in retrospective analyses [8]. Indeed, in some situations, one class of samples might occur rarely in the dataset. Then, a model might not have enough samples of such a rare class to train with. Consequently, the model might lack the ability to detect this class of samples. In this case, the model's performance score might appear to be high, to the expense of the small proportion of misclassified samples that belong to the rare class. Instead, class-wise accuracy (classification accuracy within a specific class) should be measured to fully understand the model's performance [8].

Lastly, radiomics studies conducted on retrospective data only provide a proof of concept and a low level of evidence for their models and biomarkers. Clinically applicable models and biomarkers must be examined within prospective studies. Without the high level of evidence provided by randomized trials, it would be difficult to convince clinicians to incorporate radiomics into their practice.

## Conclusions

To summarize, AI has the potential to improve many facets of SBRT-treated lung cancer. Whether AI is used for the discovery of prognostic and predictive biomarkers or the creation of algorithmic models, radiomics has the potential to change the field of pulmonary oncology by providing tools and support systems for healthcare professionals. Many research groups around the world are trying to improve and fine-tune the performance of algorithmic models that could automate and personalize the delivery of SBRT. While many attempts have yet to attain the standard required for clinical use, the advancements in the field are encouraging and promising. With the increasing amount of data, the continuous improvement of technology and the increased awareness of AI as a tool in medicine, it is only a matter of time until doctors are able to accurately predict the success of SBRT in NSCLC patients.
